# Metabolomics Combined with Physiology and Transcriptomics Reveal the Response of *Samsoniella hepiali* to Key Metabolic Pathways and Its Degradation Mechanism during Subculture

**DOI:** 10.3390/antiox13070780

**Published:** 2024-06-27

**Authors:** Hui He, Zhengfei Cao, Tao Wang, Chuyu Tang, Yuling Li, Xiuzhang Li

**Affiliations:** State Key Laboratory of Plateau Ecology and Agriculture, Qinghai Academy of Animal and Veterinary Science, Qinghai University, Xining 810016, China; he15226330573@163.com (H.H.); c1474477969@163.com (Z.C.); 13085500761@163.com (T.W.); chuyutang0410@163.com (C.T.)

**Keywords:** strain degradation, metabolites, *Samsoniella hepiali*, transcriptomics, antioxidant activity

## Abstract

During the subculture of filamentous fungi, obvious signs of degradation occur which affect the growth and development of the strain, change the content of metabolites, and interfere with gene expression. However, the specific molecular mechanism of filamentous fungi degradation is still unclear. In this study, a filamentous fungus *Samsoniella hepiali* was used as the research object, and it was continuously subcultured. The results showed that when the strain was subcultured to the F8 generation, the strain began to show signs of degradation, which was manifested by affecting the apparent morphology, reducing the growth rate and sporulation, and destroying the antioxidant system. Further transcriptome and metabolomics analyses were performed, and the results showed differentially expressed genes (DEGs) and differentially accumulated metabolites (DAMs) that were mainly enriched in four metabolic pathways: ABC transporters; fatty acid degradation; alanine, aspartate, and glutamate metabolism; and purine metabolism. Many of the metabolites that were significantly enriched in different pathways may mainly be regulated by genes belonging to proteins and enzymes, such as *Abcd3*, *Ass1*, and *Pgm1*. At the same time, in the process of subculture, many genes and metabolites that can induce apoptosis and senescence continue to accumulate, causing cell damage and consuming a lot of energy, which ultimately leads to the inhibition of mycelial growth. In summary, this study clarified the response of *S. hepiali* strains to key metabolic pathways during subculture and some reasons for the degradation of strains.

## 1. Introduction

Filamentous fungi are a class of microorganisms widely distributed on land and in the ocean. They can secrete a large number of active secondary metabolites during their growth and development. Human beings have successfully synthesized many valuable enzymes and natural products by using these metabolites [1,2]. For example, gibberellic acids compounds, which are widely used in the field of agricultural production, have the effect of promoting plant growth. They are mainly derived from the filamentous fungus *Fusarium fujikuroi* [3]. However, continuous subculture and strain preservation will lead to high-frequency degradation of filamentous fungi, mainly characterized by unstable phenotypic development and decreased virulence of strains, which seriously restricts its industrial production and causes huge economic losses [4,5]. Therefore, it is of great significance for future industrial large-scale production to clarify the specific molecular mechanism of filamentous fungi degradation during subculture in order to prevent its degradation.

A large number of studies have shown that the degradation of filamentous fungi can lead to changes in colony morphology [6], reduction in the number of hyphae and sporulation [7,8], reduced growth rate [8], etc., and that it has even caused some filamentous fungi to completely lose the ability to produce fruiting bodies [9]. Therefore, the degradation of strains seriously affects the yield and quality of filamentous fungi. In addition, degradation leads to the accumulation of a large quantity of reactive oxygen species (ROS) in the strain. At the same time, the activity of antioxidant enzymes is reduced, and the levels of exopolysaccharides, cellulases, and amylases become unbalanced, which affects the normal physiological process of the strain and causes oxidative stress [9,10]. Further studies on the degradation of filamentous fungi have found that degradation also significantly changed the expression of the strain’s metabolic profile. For example, previous studies on a subculture of *Volvariella volvacea* showed that degradation reduced the content of polysaccharides, proteins, polyphenols, and flavonoids in the strain [11]; a study on a degenerated *Cordyceps militaris* strain further showed that the degradation of the strain resulted in a significant decrease in the content of various metabolites in the mycelium compared to the normal strain, especially the content of cordycepin [12]. Relatedly, the degradation of filamentous fungi is usually accompanied by changes in gene expression. Chang et al.’s research on *Fusarium oxysporum* f. sp. *niveum* showed that degradation led to a significant decrease in the expression levels of *Gas1*, G-protein β subunit *Fgb1*, mitochondrial protein *Fow1*, and pH response transcription factor *PacC* genes in the mycelium that are closely related to the virulence of the strain. Among them, the *Gas1* gene is necessary for cell wall biosynthesis and morphogenesis in some Fusarium fungi [4]. These studies provide a basis for the study of the degradation of filamentous fungi, but the specific molecular mechanism is still unclear. Therefore, further exploration of the changes to genes and metabolites in the mycelium of normal and degraded strains during the process of successional culture is important to understanding the molecular mechanism of senescence produced by the strains.

*Samsoniella hepiali* is a medicinal fungus isolated from fresh *Cordyceps sinensis*, and previous studies have shown that its active ingredients and pharmacological effects are similar to those of natural *Cordyceps sinensis*. Therefore, *S. hepiali* is considered to be an effective substitute for natural *Cordyceps sinensis*, which alleviates the dilemma of the depletion of natural *Cordyceps sinensis* resources [13,14,15]. It is reported that fungi are usually used for large-scale industrial production because they are suitable for growth in various substrates and have multi-functional characteristics. However, the degradation of strains during passage or preservation causes huge economic losses [16]. Early studies on filamentous fungi such as *Cordyceps militaris* and *Metarhizium anisopliae* suggest that the causes of degeneration may be related to changes in mating type, gene mutations, accumulation of intracellular toxic and harmful substances, and oxidative damage to strains [17,18]. *S. hepiali* is similar to other fungi in the process of passage, and obvious degradation of the strain occurs. However, the specific molecular mechanism of this degradation is still unclear. Therefore, in this study, physiological, transcriptomic, and non-targeted metabolomics methods were used to analyze the response of strains to senescence or degradation during subculture in order to provide a theoretical reference for the industrial large-scale production of *S. hepiali* and the prevention of its degradation.

## 2. Materials and Methods

### 2.1. Samples

The original strains were provided by the *Cordyceps sinensis* Laboratory of the Qinghai Academy of Animal and Veterinary Science, Qinghai University. Different generations of strains were continuously subcultured using PDA (potato dextrose agar, PDA) medium. The preserved original strain was recorded as the F0 generation. The F0 generation was transferred to the center of the PDA plate medium and sealed with a sealing membrane, and this constituted the first generation, recorded as F1, with 30 replicates per generation. After 7 days of growth, a small colony in the PDA medium was selected to continue and transfer to the next generation and so on for continuous subculture. Finally, a total of 11 generations of strains were obtained. All strains were placed in a constant temperature culture room at 18 °C for dark culture. F1, F8, and F11 strains were selected for subsequent metabolomics and transcriptomics analysis.

### 2.2. Observation of Apparent Morphology and Determination of Growth Rate, Dry Weight, Fresh Weight, and Sporulation

Apparent morphology: After 35 days of culturing, the growth of different generations of strains was photographed and recorded.

Growth rate: After 35 days of culturing of different generations of strains, three dishes with good growth conditions were selected. Their diameters were measured by cross method, and the average value was taken.

Dry and fresh weights: The culture medium with mycelium was removed from the petri dish after 35 d of strain cultivation; the complete *S. hepiali* colony was obtained after scraping the medium, and the fresh weight of mycelium was obtained by weighing using an electronic balance. The colonies were dried to constant weight at 45 °C using a blast drying oven [19], and the dry weight of mycelium was obtained by weighing with an electronic balance. Three replicates were set for each group.

Sporulation: After 35 d of culturing of different generations of strains, a stopper borer 6 mm in diameter was used to select the spore-producing parts from the center of the colony to halfway to the edge for perforation and sampling, and then samples were placed in 10 mL of 0.05% Tween-80 sterile water, and the spores were fully eluted and mixed with a vortex oscillator and finally filtered through sterilized gauze to obtain the spore suspension. The spore production was calculated by hemocytometer, and the average value was taken from 3 petri dish replicates. The calculation formula was:

Sporulation (number/mL) = Total number of spores in 80 squares/80 × 400 × 10^4^ × dilution multiple

### 2.3. Antioxidative Enzyme Activity and Malondialdehyde (MDA) Content

The test kits were purchased from Suzhou Keming Biotechnology Co., Ltd. (Comin, Suzhou, China). Superoxide dismutase (SOD), catalase (CAT), and glutathione peroxidase (GSH-Px) were selected as antioxidant enzyme indicators. Measurements were taken following the method described for the kit. Malondialdehyde (MDA) was measured following the method of the MDA content kit. Total antioxidant capacity (T-AOC) was measured by the total antioxidant capacity (DPPH method) kit.

### 2.4. Untargeted Metabolomics Analysis

The samples were weighed before metabolite extraction. The freeze-dried powder was ground at 65 Hz for 1 min in a 2 mL Eppendorf tube containing 5 mm tungsten beads. Metabolites were extracted using a 1 mL precooled mixture of methanol, acetonitrile, and water (*v*/*v*/*v*, 2:2:1) and were then placed in an ice bath for 1 h for ultrasonic oscillation. Subsequently, the mixture was placed at −20 °C for 1 h and centrifuged at 14,000× *g* at 4 °C for 20 min. The supernatant was recovered in a vacuum and concentrated to dryness.

A UPLC-ESI-Q-orbitrap-MS system (UHPLC, Shimadzu Nexera X2 LC-30AD, Shimadzu, Japan) and Q-Exactive Plus (Thermo Scientific, San Jose, CA, USA) were used for metabolomics analysis. For liquid chromatography (LC) separation, samples were analyzed on an ACQUITY UPLC and HSS T3 column (2.1 × 100 mm, 1.8 μm) (Waters, Milford, MA, USA). Mobile phase A: 0.1% FA aqueous solution; B: 100% acetonitrile (ACN). The flow rate was 0.3 mL/min. The gradient was 0% buffer B for 2 min, increased linearly to 48% within 4 min, increased to 100% within 4 min and maintained for 2 min, then decreased to 0% buffer B within 0.1 min, and the rebalance period was 3 min. The HESI source conditions were set as follows: spray voltage—3.8 kv (positive) and 3.2 kv (negative); capillary temperature—320 °C; sheath gas (nitrogen) flow—30 arb (any unit); auxiliary gas flow—5 ARB; probe heater temperature—350 °C; s-lens rf level—50. The instrument was set to collect full MS in the range of *m*/*z* 70–1050 Da. The full MS scan resolution was 70,000 (*m*/*z* 200), and the MS/MS scan resolution was 17,500 (*m*/*z* 200). The maximum injection time of MS was 100 ms, the maximum injection time of MS/MS was 50 ms, the separation window of MS2 was 2 *m*/*z*, and the normalized collision energy (step) of fragmentation was 20, 30, and 40. Quality control (QC) samples were prepared by mixing all of the representative samples to be tested in equal proportions and were used for data standardization. During the collection process, blank samples (75% ACN) and QC samples were injected into every 6 samples.

MS-DIAL was used to perform peak alignment, retention time correction, and peak area extraction on the original mass spectrometry data. Metabolites were identified by mass (mass tolerance < 10 ppm) and MS/MS data (mass tolerance < 0.02 Da), which were accurately matched with public databases such as HMDB, mass bank, and our self-built metabolite standard library. Among the extracted ion features, only variables with more than 50% non-zero measurements in at least one set were retained.

All multivariate data analyses and modeling were performed using R (version: 4.0.3) and R packages (https://CRAN.R-project.org/package=pheatmap, accessed on 15 January 2024) [20,21]. The data were mean-centered using Pareto scaling. Principal component analysis (PCA), orthogonal partial least squares discriminant analysis (PLS-DA), and partial least squares discriminant analysis (OPLS-DA) were used to establish the model. All of the evaluated models were tested for over-fitting by the method of permutation test. The descriptive performance of the model was determined by the values of R2X (cumulative) (perfect model: R2X (cum) = 1) and R2Y (cumulative) (perfect model: R2Y (cum) = 1), and the predictive performance was measured by Q2 (cumulative) (perfect model: Q2 (cum) = 1) and permutation test (n = 200). The permutation model should not be able to predict the category; the R2 and Q2 values at the y-axis intercept must be lower than the R2 of the Q2 and non-permutation models. OPLS-DA allows the use of variable importance in projection (VIP) to determine differential metabolites. The VIP score represents the contribution of a variable to the discrimination between samples of all categories. Mathematically, these scores are calculated as the weighted sum of squares of PLS weights for each variable. The average VIP value is 1. Usually, a VIP value greater than 1 is considered significant. A high score is consistent with a strong ability to distinguish, so it constitutes the standard for biomarker selection. The statistically significant variable projection influence threshold (VIP) values obtained by the OPLS-DA model and the two-tailed Student’s *t*-test (*p*-value) of the univariate analysis level normalized raw data were used to obtain differential metabolites. One-way analysis of variance (ANOVA) was used to calculate the *p*-value, and multiple sets of analyses were performed. Metabolites with VIP values greater than 1.0 and *p*-values less than 0.05 were considered statistically significant metabolites. The folding change was calculated as the logarithm of the average mass response (area) ratio between two arbitrary classes. On the other hand, the identified differential metabolites were clustered by R package [20]. Among them, the clustering data and correlation were based on the mass spectrometry intensity values of the differential metabolites in the comparison group.

To determine the biological pathways that were interfered with, KEGG pathway analysis was performed on the differential metabolite data using the KEGG database (http://www.kegg.jp, accessed on 8 January 2024). KEGG enrichment analysis was performed using Fisher’s exact test and FDR correction for multiple tests. The enriched KEGG pathway was nominally statistically significant at the *p* < 0.05 level.

### 2.5. Transcriptome Analysis

Total RNA was isolated using Trizol Reagent (Invitrogen Life Technologies, Carlsbad, CA, USA), and then concentration, quality, and integrity were measured using a nanodrop spectrophotometer (Thermo Scientific, Waltham, MA, USA). For RNA sample preparation, 3 μg RNA was used as the input material. TruSeq RNA sample preparation kits (Illumina, San Diego, CA, USA) were used to generate sequencing libraries. First, mRNA was purified from total RNA using poly-T oligo magnetic beads. Pyrolysis was conducted at high temperatures using divalent cations in Illumina’s proprietary fragmentation buffer. The first-strand cDNA was synthesized using random oligonucleotides and Super Script II. Subsequently, DNA Polymerase I and RNase H were used for second-strand cDNA synthesis. Through exonuclease/polymerase activity, the remaining overhang was converted into a flat end, and the enzyme was removed. After adenylation of the 3′ end of the DNA fragment, the Illumina PE linker oligonucleotide was ligated to prepare for hybridization. An AMPure XP system (Beckman Coulter, Beverly, CA, USA) was used to purify the library fragments, and cDNA fragments between 400 bp and 500 bp were selected. In a 15-cycle PCR reaction, Illumina PCR Primer Cocktail was used to selectively enrich DNA fragments that connect adaptor molecules at both ends. After purification, the product was quantified on the Bioanalyzer 2100 system (Agilent, Santa Clara, CA, USA) using Agilent’s high-sensitivity DNA assay. The sequencing library was sequenced on the Nova Seq 6000 platform (Illumina) produced by Shanghai Bioprofile Technology Company, Ltd. (Bioprofile, Shanghai, China).

The samples were sequenced on the platform to obtain image files, which were transformed by the software of the sequencing platform to generate raw data (Raw Data) in the FASTQ format. The sequencing data contained a large number of adaptors and low-quality reads, so we used fast (0.22.0) software to filter the sequencing data to obtain high-quality sequences (clean reads) for further analysis. For transcriptome sequencing projects without reference genomes, we used Trinity (v2.15.1) software to perform a clean reads montage of transcripts for later analysis. After the completion of the montage operation, the FASTA format transcript sequence file could be obtained, and the longest transcript of each gene was extracted as the representative sequence of the gene, named as an independent gene. We annotated the gene function of independent genes. The databases used for gene function annotation included NR (NCBI non-redundant protein sequences), GO (Gene Ontology), KEGG (Kyoto Encyclopedia of Genes and Genome), eggNOG (Evolutionary Genealogy of Genes: Non-supervised Orthologous Groups), Swiss-Prot, and Pfam. RSEM (v2.15) was used to statistically compare the read count values on each gene as the original expression level of the gene, and FPKM was used to standardize the expression level. Next, we used DESeq (v1.38.3) to analyze differentially expressed genes, and the screening conditions were expression difference multiple |log2FoldChange| > 1 and significant *p*-value < 0.05. At the same time, the R language Pheatmap (v1.0.12) software package was used to perform a two-way cluster analysis of all differential genes in the sample. According to the expression level of the same gene in different samples and the expression pattern of different genes in the same sample, the Euclidean method was used to calculate the distance, and the complete linkage method was used to cluster to obtain the heat map. Cluster analysis was performed using the FPKM values of the differential genes in the comparison group, and correlation analysis was performed using the FPKM values of all genes. All genes were mapped to terms in the Gene Ontology database, and the number of differentially enriched genes in each term was calculated. GO enrichment analysis of differential genes (all DEGs/up DEGs/down DEGs) was performed using top GO (v2.50.0). The *p*-value was calculated by the hypergeometric distribution method (the significant enrichment standard was *p*-value < 0.05), and the GO term of significant enrichment of differential genes was found to determine the main biological functions performed by differential genes. KEGG pathway enrichment analysis of differential genes was performed using the ClusterProfiler (v4.6.0) software, focusing on significant enrichment pathways with *p*-value < 0.05. The Varscan (v2.3.9) software was used to obtain SNP and InDel sites, and the filtering criteria were (1) SNP site base Q > 20; (2) the number of reads covering the site > 8; (3) the number of reads supporting the mutation site > 2; (4) the *p*-value of SNP locus < 0.01. The MISA (v1.0) software was used to search for SSR sites in the sequence. The parameters used are as follows: the minimum number of repeats of 1–6 bases was 10, 6, 5, 5, 5, and 5, respectively. The distance between the two SSRs was less than 100 bp. Transdecoder (v5.7.0) software was used to predict the ORF (open reading frame) of independent gene sequences. Transcription factor prediction was used to compare plants and animals with the Plant TFDB (Plant Transcription Factor Database) and the Animal TFDB (Animal Transcription Factor DataBase) databases, respectively, to predict the transcription factors and family information of the transcription factor. Then, the DEGs predicted as transcription factors were counted. The STRING database (https://string-db.org/, accessed on 24 January 2024) was used for protein interaction analysis to reveal the relationship between target genes.

### 2.6. Quantitative Real-Time Polymerase Chain Reaction (qRT-PCR) Verification

To validate the RNA-seq data, we selected 15 genes associated with ABC transporters; fatty acid degradation; alanine, aspartate, and glutamate metabolism; and purine metabolism for qRT-PCR validation. The primers used for qRT-PCR were shown in Table S1.

There are generally three bands in the complete RNA electrophoresis map, which are 28S, 18S, and 5S rRNA, from top to bottom. The purpose of electrophoresis is to detect the integrity of 28S and 18S. If all three bands exist, and the main band is clear, single, and bright, then RNA is considered to be good. After the synthesis of the first strand of cDNA, fluorescence quantitative PCR reaction and real-time PCR reaction were performed.

## 3. Results

### 3.1. Growth Rate, Fresh Weight, Dry Weight and Sporulation

**Figure 1:**
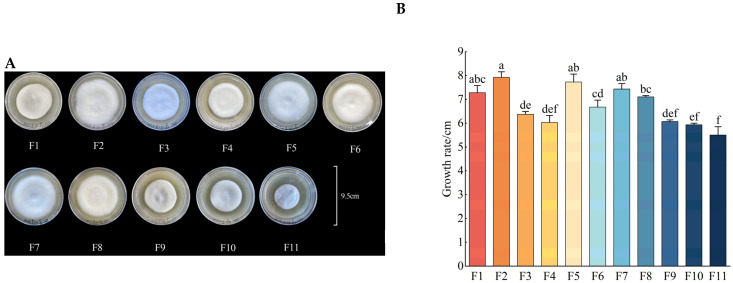

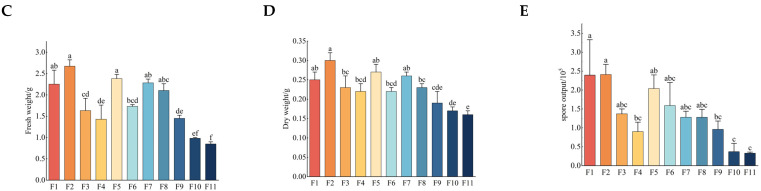
The changes to the (**A**) apparent morphology, (**B**) growth rate, (**C**) fresh weight, (**D**) dry weight, and (**E**) sporulation of different generations of strains after 35 days of culturing. Different letters above the columns indicate significant difference (*p* < 0.05).

After 35 days of culture, the growth rate, fresh weight, and dry weight of the strains did not show obvious regularity before the F8 generation, but following the F8 generation, the growth rate, fresh weight, and dry weight of the strains of each generation began to show a significant downward trend and reached their lowest levels in the F11 generation, indicating that the strains may begin to show obvious signs of degradation starting at the F8 generation (Figure 1A–D). The growth rate, fresh weight, and dry weight of the F2 generation reached their highest levels out of the total of 11 generations, these levels being 1.44, 3.14, and 1.88 times the values from the F11 generation, respectively. In addition, the sporulation of different generations of strains showed a downward trend in general, and it also reached its highest level in the F2 generation, which was significantly different from the value of the F11 generation (*p* < 0.05). The sporulation of the F2 generation was 7.3 times that of the F11 generation (Figure 1E).

### 3.2. Antioxidant Activity and Malondialdehyde (MDA) Content

**Figure 2:**
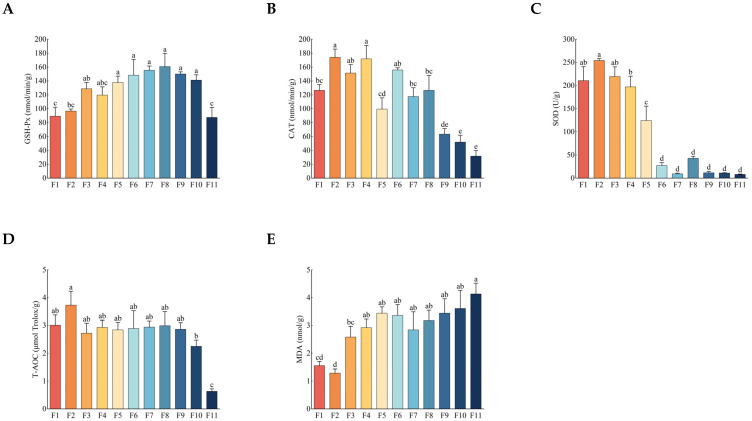
Changes in the (**A**) GSH-Px, (**B**) CAT, (**C**) SOD, (**D**) T-AOC, and (**E**) MDA in different generations of strains after 35 days of culturing. Different letters above the columns indicate significant difference (*p* < 0.05).

With the increase of culture generations, the contents of glutathione peroxidase (GSH-Px), catalase (CAT), superoxide dismutase (SOD), and the total antioxidant capacity (T-AOC) in different generations of strains began to decrease gradually from the F8 generation, while the content of malondialdehyde (MDA) began to increase gradually from the F8 generation. The lowest and highest levels were reached in the F11 generation, but there was no obvious change in the strains before the F8 generation (Figure 2). The trend of antioxidant activity and MDA content in different generations of strains was similar to those of growth rate, fresh weight, dry weight, and sporulation. This further indicated that the subculture strains began to show obvious signs of degradation from the F8 generation.

### 3.3. Metabolomic Analysis

#### 3.3.1. Metabolic Analysis of Different Generations of Subcultured Strains

**Figure 3:**
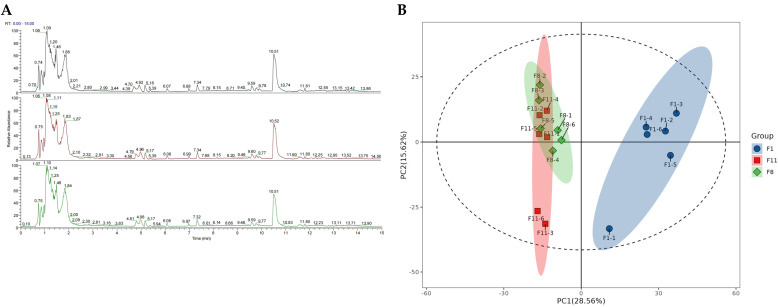
The (**A**) total ion chromatographic diagram (TIC) and (**B**) PCA first and second principal component scatter plots of the three groups of samples. In (**B**), each point represents a sample, and the samples in the same group are represented by the same color.

As the different generations of strains started to show obvious signs of degradation at the F8 generation, there were significant differences in most physiological indicators between them and the F1 and F11 generations. We therefore performed sequencing analyses of the F1, F8, and F11 generations to clarify the changes in metabolites between the three different generations of strains. The QC samples of F1, F8, and F11 were detected using UPLC-Q-Exactive Plus MS to verify repeatability and stability (Figure 3A). The results showed that the response intensities and retention times of each chromatographic peak were basically the same, indicating that the variation caused by instrument error during the experiment was small and that the data quality was reliable. In addition, the detected metabolites were annotated based on multiple search databases. The results showed that a total of 1529 metabolites were identified from the three groups of samples, including 936 positive ion mode metabolites (ESI+) and 593 negative ion mode metabolites (ESI−) (Table S2).

Principal component analysis (PCA) is an unsupervised data analysis method. Principal component analysis of metabolites is helpful for understanding the variability between and within the sample groups. PCA results showed that all of the samples were divided into three groups, indicating that there were differences in metabolic profiles between different generations. However, there was an overlap between the F8 and F11 generations of samples in the PCA, which may be due to the similarity of the metabolites and their content levels in the samples of the two generations, suggesting that there is a certain degree of similarity between the metabolic profiles of the degraded strains. In addition, the first principal component and the second principal component explained 44.18% of the total variability of the data set. Among them, the F1 samples were on the positive X axis, while the F8 and F11 samples were on the negative X axis, indicating that the metabolites of different generations of samples changed significantly with the degradation of the strain (Figure 3B). The results showed that there were significant differences in the metabolic profiles between normal strains and degraded strains.

#### 3.3.2. Identification of Differentially Accumulated Metabolites (DAMs) in Different 

##### Subculture Strains

**Figure 4:**
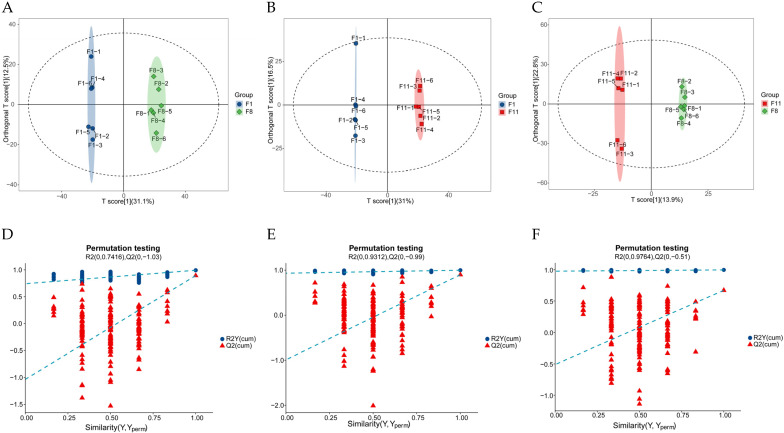

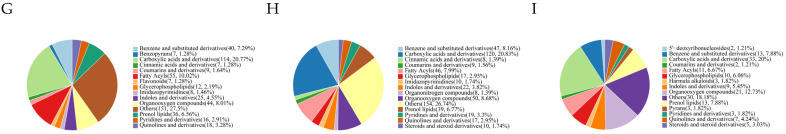
(**A**,**D**,**G**;**B**,**E**,**H**;**C**,**F**,**I**) denote OPLS-DA score plots, OPLS-DA substitution test plots, and metabolite HMDB Class classification ring plots for F1 vs. F8, F1 vs. F11, and F8 vs. F11, respectively.

Orthogonal partial least squares discriminant analysis (OPLS-DA) is a supervised discriminant analysis statistical method which is helpful for distinguishing DAMs of different samples. The OPLS-DA score plot was obtained through pairwise comparison of the OPLS-DA model. Similar to the results of PCA, the three groups of different samples were obviously separated (Figure 4A–C). In addition, the OPLS-DA model obtained after 200 permutation tests showed that R^2^X > 0.4 between all pairwise comparison groups, the values of R^2^Y and Q^2^ were higher (F1 vs. F8, R^2^X(cum) = 0.436, R^2^Y(cum) = 0.993, Q^2^(cum) = 0.893; F1 vs. F11, R^2^X(cum) = 0.588, R^2^Y(cum) = 0.998, Q^2^(cum) = 0.897; F8 vs. F11, R^2^X(cum) = 0.455, R^2^Y(cum) = 0.997, Q^2^(cum) = 0.671), and the model did not overfit (Figure 4D–F) indicating that the OPLS-DA model has high predictability and reliability and can effectively screen differential metabolites. DAMs were screened using multivariate or univariate statistical analyses with the variable importance for the projection (VIP) > 1 and the *p*-value < 0.05. The results showed that there were 573 DAMs (253 up-regulated and 320 down-regulated) (Table S3) between F1 and F8; there were 598 DAMs (303 up-regulated and 295 down-regulated) between F1 and F11 (Table S4); and there were 173 DAMs (109 up-regulated and 64 down-regulated) between F8 and F11 (Table S5). Among them, carboxylic acids and derivatives (20~20.83%), organooxygen compounds (8.01~12.73%), benzene and substituted derivatives (7.29~8.16%), fatty acyls (6.67~10.02%), and prenol lipids (6.56~7.88%) accounted for a large proportion in all comparison groups (Figure 4G–I).

**Figure 5:**
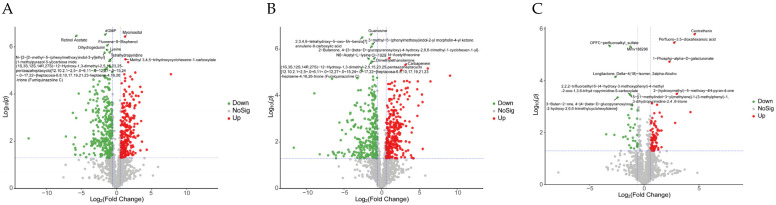
Volcano diagrams of (**A**) F1 vs. F8, (**B**) F1 vs. F11, and (**C**) F8 vs. F11. Green means down, and red means up.

The volcano plot drawn using the univariate analysis method can intuitively show the significance of DAMs changes between the two groups of samples, which is helpful for screening potential marker metabolites. In the F1 vs. F8 comparison group, 10 DAMs were significantly up-regulated or down-regulated (two up-regulated and eight down-regulated). Among them, myoinositol can reduce the risk of gestational diabetes by increasing insulin sensitivity [22], which was significantly up-regulated in the F1 vs. F8 comparison group (Figure 5A). In the F1 vs. F11 comparison group, two DAMs, N-acetylthreonine and carbapenem, were significantly up-regulated, and eight DAMs, including guanosine, were significantly down-regulated. (Figure 5B). Among them, carbapenem is one of the most commonly used and effective antibiotics with broad-spectrum antibacterial activity [23], and guanosine is a purine nucleoside with anticancer and neuroprotective effects [24,25] (Figure 5B). In the F8 vs. F11 comparison group, four significantly up-regulated DAMs and six significantly down-regulated DAMs (Figure 5C) were found, which also included some bioactive metabolites. For example, centratherin, as a sesquiterpene lactone, has been shown to inhibit the proliferation of tumor cells [26]. The results showed that the changes to DAMs in different generations of strains were related to subculture generations and strain degradation or aging, so the anti-aging ability of strains could be enhanced by adjusting the content of related metabolites.

**Figure 6:**
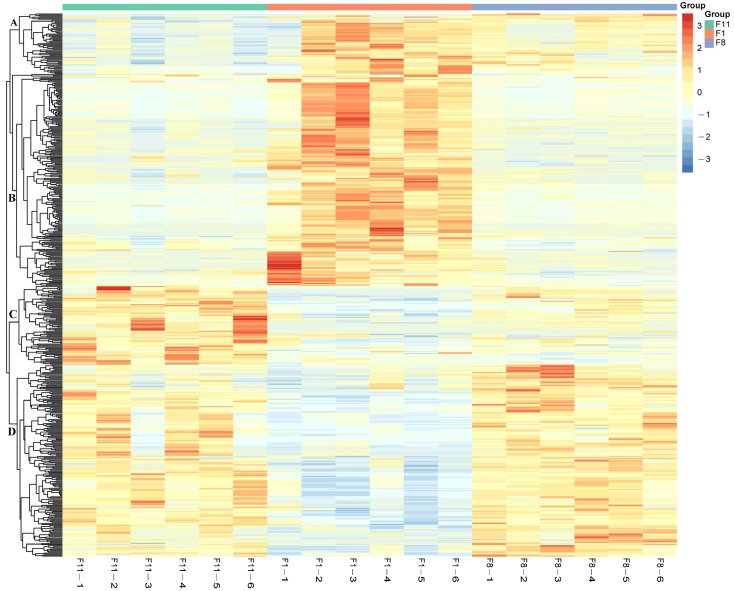
Hierarchical clustering analysis of DAMs among three different generations of samples. Redder colors indicate higher relative expression and bluer colors indicate lower relative expression.

In addition, in order to evaluate the plausibility of the candidate metabolites and, at the same time, to show more comprehensively and intuitively the relationship between the samples as well as the differences in the metabolite expression patterns among the different samples, we used qualitatively significant differences in metabolite expression to perform hierarchical clustering on each group of samples (Figure 6). The results showed that the accumulation patterns of DAMs in all subcultured strains were divided into four categories. Among them, the DAMs of cluster A and cluster B showed an up-regulation trend in the F1 generation strains, and the DAMs of cluster C and cluster D showed an up-regulation trend in the F8 and F11 generation strains. This indicated that the DAMs of different generations of strains changed greatly.

#### 3.3.3. KEGG Enrichment Analysis of DAMs

**Figure 7:**
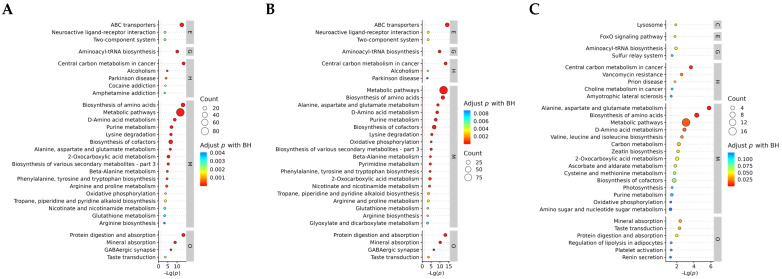
The KEGG pathway enrichment bubble maps (top 30) of group (**A**) F1 vs. F8, (**B**) F1 vs. F11, and (**C**) F8 vs. F11 were compared. Level 1 pathway classification: metabolism (M), genetic information processing (G), environmental information processing (E), cellular processes (C), organismal systems (O), human diseases (H), and drug development (D).

Through the analysis of KEGG enrichment pathways, the key metabolic pathways of different subculture strains can be identified. A total of 98 key metabolic pathways were involved in the expression in three different generations of strains (Table S6). In order to further clarify the differences in metabolic pathways between the comparison groups, we analyzed the metabolic pathways that enriched the significant top 30 (Figure 7A–C). The results showed that metabolic pathways were significantly enriched in all comparison groups. In addition, ABC transporters, aminoacyl-tRNA biosynthesis, central carbon metabolism in cancer, and biosynthesis of amino acids were significantly enriched in the F1 vs. F8 and F1 vs. F11 comparison groups. In F8 vs. F11, three metabolic pathways were significantly enriched: central carbon metabolism in cancer, biosynthesis of amino acids, and alanine, aspartate, and glutamate metabolism.

### 3.4. Transcriptomic Analysis

#### 3.4.1. Transcriptome Analysis of Different Subculture Strains

**Figure 8:**
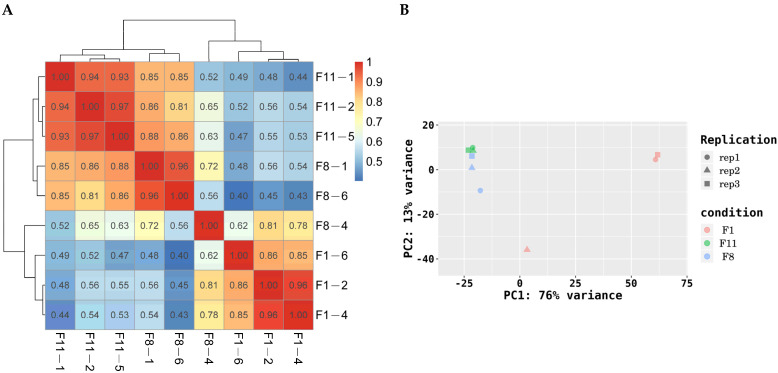
(**A**) Sample correlation test. The left and upper sides are the sample clustering, the right and lower sides in the figure are the sample names, and the squares of different colors represent the correlation between the two samples. (**B**) PCA analysis. The abscissa is the first principal component, and the ordinate is the second principal component. Different shapes in the figure represent different samples, and different colors represent different groups.

Sample libraries of three different generations of strains were constructed and sequenced. After removing low-quality reads, a total of 527,575,378 clean reads and 79,509,049,141 bp clean bases were obtained. The GC content is between 55.57~56.44%, the percentages of Q20 and Q30 are more than 98.79% and 96.44%, respectively, and the potential error rate is lower than 0.007917% (Table 1). This shows that the quality of the sequencing data is high and that they can be used for further analysis. The correlation of gene expression levels between samples is an important indicator for testing the reliability of the experiment. We used the Pearson correlation coefficient to represent the correlation of gene expression levels between samples. The results showed that the correlation coefficient between the F8 and F11 generations of the degraded strains was higher, which was greater than 0.8 in most cases. The correlation coefficient between the F1 generation and the degraded strains (F8 and F11) was less than 0.8 in most cases (Figure 8A). Principal components analysis (PCA) showed that there was a close clustering between the degenerated strains (F8 and F11). The first principal component and the second principal component clearly distinguished the normal strains (F1) and the degenerated strains (F8 and F11) (Figure 8B). This shows that there is a stable consistency between the degraded strains, and there may be some differences in gene expression between the normal strains and the degraded strains.

#### 3.4.2. Differentially Expressed Genes (DEGs) of Different Subcultured Strains

**Figure 9:**
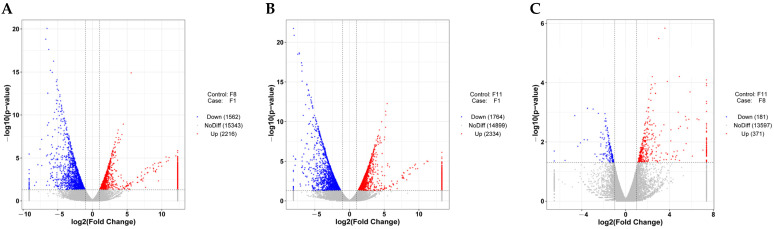
Volcano maps of differentially expressed genes in (**A**) F1 vs. F8, (**B**) F1 vs. F11, and (**C**) F8 vs. F11 comparison groups. The abscissa is log2FoldChange, and the ordinate is −log10 (*p*-value). The two vertical dashed lines in the figure are two times the threshold of expression difference; the horizontal dotted line is the *p*-value = 0.05 threshold. Red dots represent up-regulated genes in this group, blue dots represent down-regulated genes in this group, and gray dots represent non-significant differentially expressed genes.

**Figure 10:**
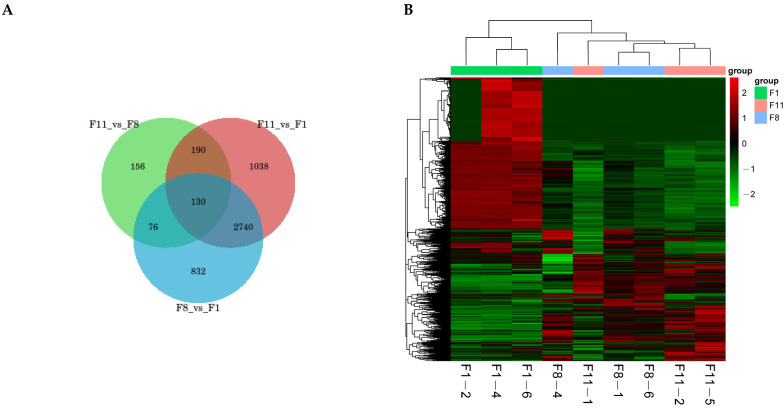
(**A**) Common and unique differential genes between the comparison groups. The sum of the numbers in each circle represents the total number of differential genes in the comparison group, and the overlapping part of the circle represents the common differential genes between the two comparison groups. (**B**) Cluster analysis of differentially expressed genes. Genes are represented horizontally, and each column is a sample. Red represents high-expressed genes, and green represents low-expressed genes.

Based on the expression difference fold|log2FoldChange|>1 and the significance *p*-value < 0.05, we analyzed the gene expression profiles of three different generations. The results showed that the number of DEGs between normal strains and degraded strains (F1 vs. F8 and F1 vs. F11) and between the degraded strains (F8 vs. F11) were very similar (Figure 9A–C). In the F1 vs. F8 comparison group, compared with F8, a total of 3778 DEGs were identified from the F1 generation strains, including 2216 up-regulated genes and 1562 down-regulated genes (Figure 9A). In the F1 vs. F11 comparison group, a total of 4098 DEGs were identified from the F1 strains, including 2334 up-regulated genes and 1764 down-regulated genes (Figure 9B). In the comparison of the degenerated strains (F8 vs. F11), only 371 up-regulated and 181 down-regulated genes were identified from the F8 generation compared to the F11 generation, with a total of 552 DEGs (Figure 9C). The number of differential genes between the comparison groups was shown by drawing a Venn diagram. The results showed that there were 832, 1038, and 156 unique and 130 common DEGs in the three different comparison groups (Figure 10A). In addition, we clustered according to the expression level of the same gene in different samples and the expression pattern of different genes in the same sample. The results showed that the samples were obviously clustered into two categories, specifically, the F1 generation was clustered into one category, while the F8 and F11 generations were clustered into one category, which further illustrated the differences in the differentially expressed gene profiles between normal strains and degraded strains (Figure 10B).

#### 3.4.3. GO Analysis and KEGG Pathway Analysis

**Figure 11:**
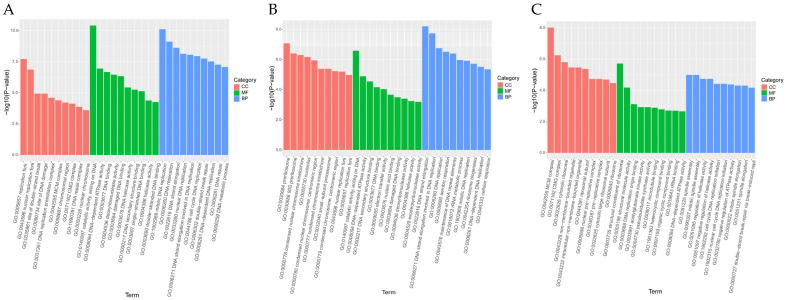
GO enrichment analysis histogram. (**A**) F1 vs. F8, (**B**) F1 vs. F11, (**C**) F8 vs. F11. The abscissa is the term of GO level 2, and the ordinate is the enriched −log10 (*p*-value) of each term.

**Figure 12:**
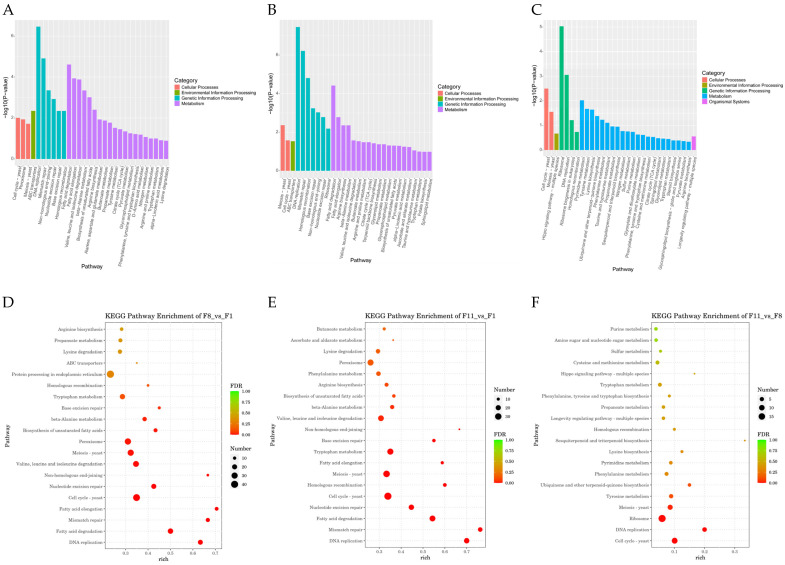
KEGG enrichment analysis. (**A**,**B**,**C**) KEGG pathway enrichment results histogram, abscissa for the pathway name, and ordinate for each pathway enrichment −log10 (*p*-value). (**A**) F1 vs. F8; (**B**) F1 vs. F11; (**C**) F8 vs. F11. (**D**,**E**,**F**) Significantly up-regulated and enriched KEGG pathway analysis bubble diagram. According to the results of KEGG enrichment, the degree of enrichment was measured by Rich factor, FDR value, and the number of genes enriched in this pathway. The Rich factor refers to the ratio of the number of differentially expressed genes enriched in the pathway to the number of differentially expressed genes annotated. The larger the Rich factor, the greater the degree of enrichment. The general value range of FDR is 0–1, and the closer to zero, the more significant the enrichment. The top 20 KEGG pathways with the smallest FDR value, that is, the most significant enrichment, were selected for display. (**D**) F1 vs. F8; (**E**) F1 vs. F11; (**F**) F8 vs. F11.

The DEGs of three different comparison groups were classified by GO enrichment analysis and KEGG enrichment analysis to clarify their main biological functions. GO enrichment analysis of DEGs showed that GO classification was mainly labeled as three functional categories: molecular function (MF), biological process (BP), and cellular component (CC) (Figure 11A–C), and 7774, 7990, and 3006 GO terms were enriched, respectively (Tables S7–S9). The top 10 GO term entries with the most significant *p*-value enrichment in each GO category were selected for display. The results showed that the significantly enriched GO terms in the F1 vs. F8 comparison group were mainly replication fork, catalytic activity, acting on DNA, and mitotic DNA replication; the significantly enriched GO terms in the F1 vs. F11 comparison group were mainly preribosome, catalytic activity, acting on DNA, and DNA strand elongation; MCM complex, structural constituent of ribosome, and spindle assembly were significantly enriched GO terms in the F8 vs. F11 comparison group (Figure 11A–C). In addition, the first 20 pathways with the smallest *p*-value, that is, the most significant enrichment, were selected for display to study the effect of subculture process on the DEGs enrichment pathway. The results showed that DEGs in the F1 vs. F8 comparison group were highly enriched in KEGG pathways such as fatty acid elongation, fatty acid degradation, mismatch repair, and DNA replication; DEGs in the F1 vs. F11 comparison group were highly enriched in KEGG pathways such as fatty acid degradation, homologous recombination, mismatch repair, and DNA replication; and the KEGG pathway of DEGs in the F8 vs. F11 comparison group was highly enriched in pathways such as pyrimidine metabolism, cell cycle yeast, DNA replication, and ribosome (Figure 12A–C). Based on the above KEGG enrichment results, we further analyzed the first 20 significantly enriched and up-regulated KEGG pathways by drawing bubble plots. The results showed that these significantly enriched KEGG pathways showed a significant up-regulated trend in the three different comparison groups (Figure 12D–F).

### 3.5. Joint Analysis of the Transcriptome and Metabolome

#### 3.5.1. Correlations between DEGs and DAMs

**Figure 13:**
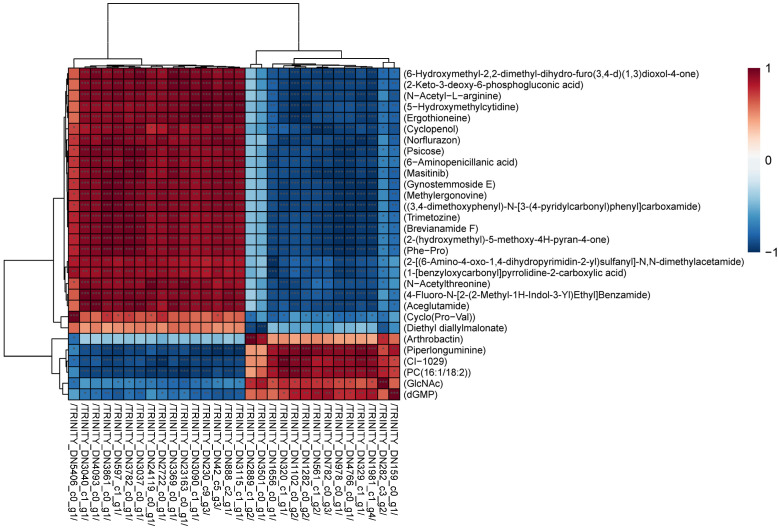
Significant correlation hierarchical clustering heat map (Top 30). Each row represents one omics element (DEGs), and each column represents another omics element (DAMs). The *** in the heat map small grid indicates the correlation test *p*-value < 0.001, ** indicates the correlation test *p*-value < 0.01, and * indicates the correlation test *p*-value < 0.05.

In order to clarify the correlation between DAMs and DEGs in three different generations during subculture, we conducted a joint analysis (Figure 13). The results showed that except diethyl diallylmalonate and arthrobactin, most of the remaining DAMs and DEGs had significant or extremely significant positive or negative correlations. Among them, the accumulation of these metabolites was mainly affected by the two gene types of protein and enzyme. This indicates that these genes may be directly or indirectly involved in the regulation of the content level of these metabolites.

#### 3.5.2. Critical Pathway Analysis

**Figure 14:**
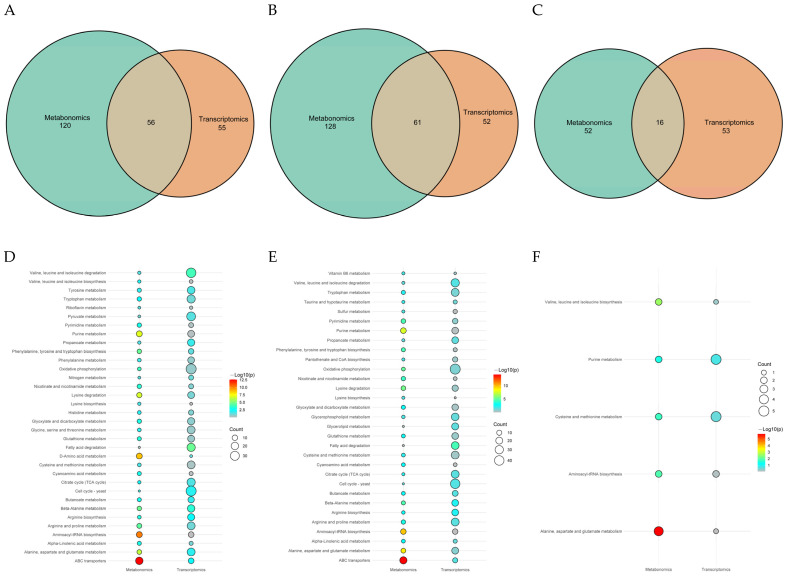
(**A**,**B**,**C**) Common KEGG pathway enrichment Venn diagram between different groups. (**A**) F1 vs. F8; (**B**) F1 vs. F11; (**C**) F8 vs. F11. (**D**,**E**,**F**) Common and significant KEGG comparative bubble plots. (**D**) F1 vs. F8; (**E**) F1 vs. F11; (**F**) F8 vs. F11.

**Figure 15:**
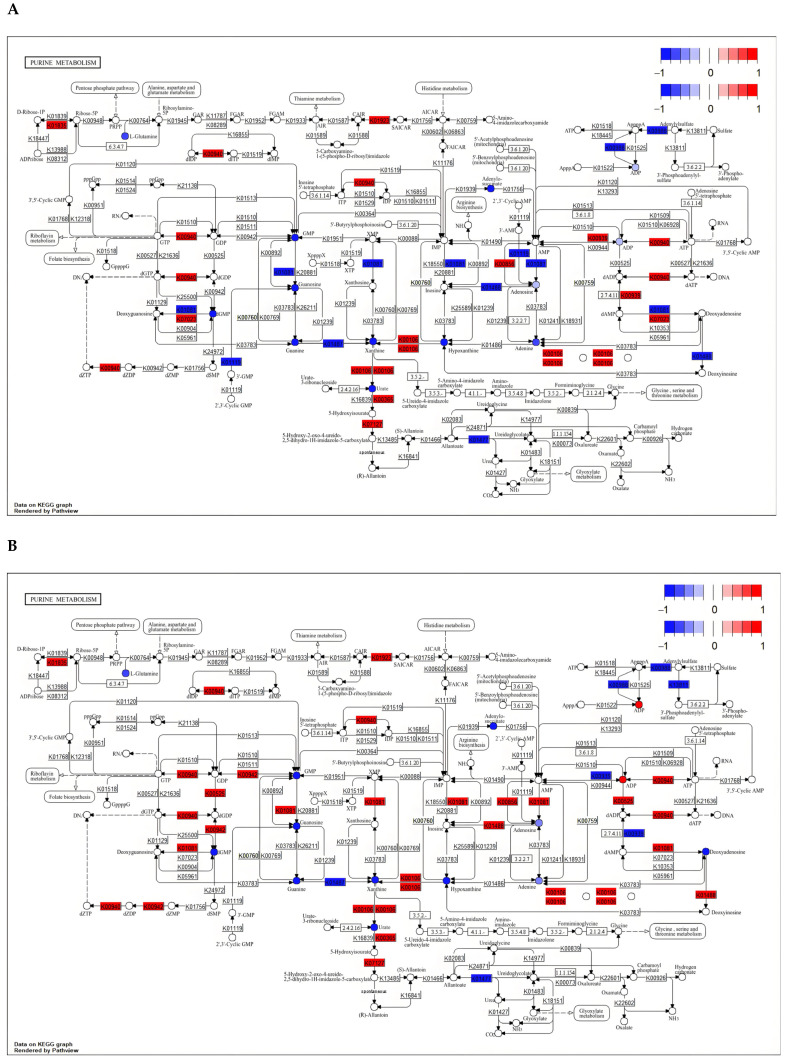
Pathway maps of DAMs and DEGs from different comparison groups (purine metabolism). Red indicates differential up-regulation, blue indicates differential down-regulation, colored dots indicate significantly different metabolites, and colored rectangles indicate significantly different genes. (**A**) F1 vs. F8; (**B**) F1 vs. F11.

In order to clarify the common pathway of the three different comparison groups, we mapped the DAMs and DEGs of these different comparison groups to the KEGG database. The results showed that 56, 61, and 16 pathways were co-enriched by DAMs and DEGs in F1 vs. F8, F1 vs. F11, and F8 vs. F11, respectively (Figure 14A–C). Further analysis of common pathways between different groups showed that DAMs and DEGs were mainly enriched in ABC transporters; fatty acid degradation; alanine, aspartate, and glutamate metabolism; and purine metabolism (Figure 14D–F and Figure 15A,B). In the ABC transporters pathway, compared with the F1 strain, the F8 and F11 strains significantly increased the accumulation of deoxycytidine and phosphoric acid, and five transcription factors (atrD, Abcb11, atrC, Abcd3, and Abcb4) were significantly up-regulated while one transcription factor (STE6) was significantly down-regulated (Tables S10 and S11). In the fatty acid degradation pathway, two transcription factors (Acsl1 and Acsl5) were significantly up-regulated in the F8 and F11 generations compared to the F1 generation (Tables S10 and S11). In the alanine, aspartate, and glutamate metabolism pathway, compared with the F1 generation, 11 transcription factors (Csad, Ass1, Gpt, Asl, Dpyd, Glud1, Aldh4a1, Abat, Got2, Gpt2, and Got1) were significantly up-regulated, and six transcription factors (GOT2, gatA, gdh-1, GFA1, GLN1, and GDH2) were significantly down-regulated in the F8 and F11 generation strains, while increasing the accumulation of oxoglutaric acid and GABA (Tables S9 and S10). In the purine metabolism pathway, eight transcription factors (Adk, Pgm1, Paics, Ak2, Ttr, Nme2, Aox3, and Uox) were significantly up-regulated, and five transcription factors (MDM34, fit1, alc-1, ada2a, and SPCC1672.03c) were significantly down-regulated in the F8 and F11 generations compared to the F1 generation strains. Among them, many purine metabolites were significantly enriched, such as hypoxanthine, xanthine, and guanosine (Tables S10 and S11). This indicates that strain degradation may be related to the enrichment of purine metabolites.

### 3.6. Confirmation of DEGs via qRT-PCR

**Figure 16:**
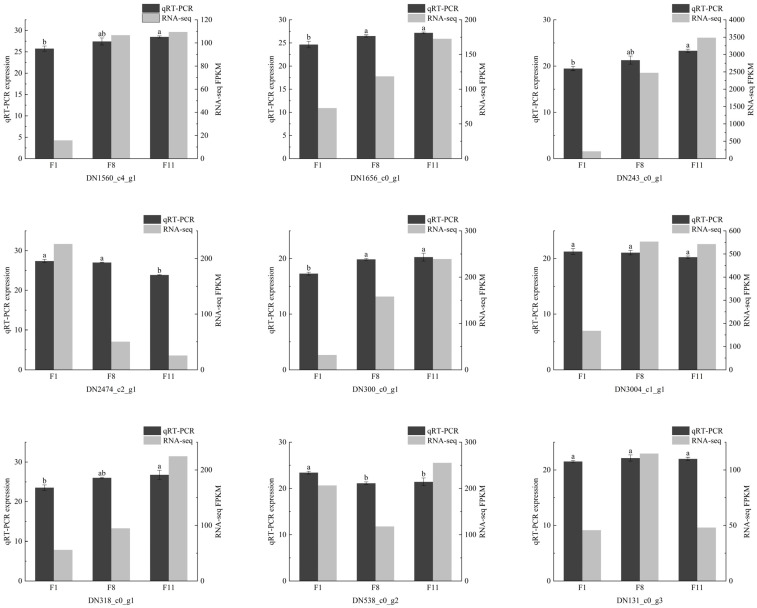

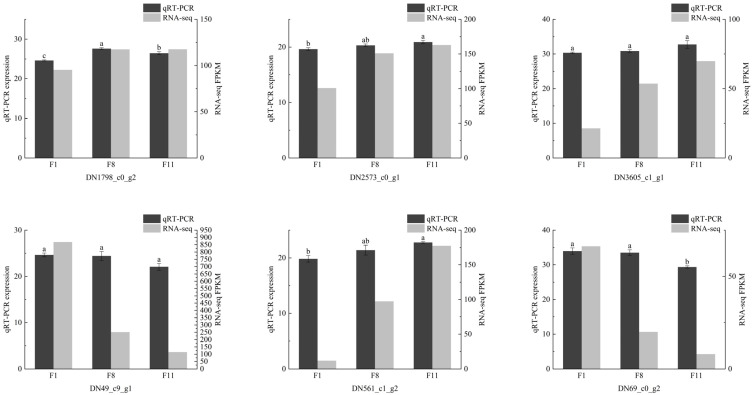
The qRT-PCR validation of 15 DEGs. The different letters on the column indicate significant differences (*p* < 0.05), and the error bar is three biologically repeated means ± SEM.

In order to verify the stability and reliability of transcriptome data, 15 DEGs related to ABC transporters; fatty acid degradation; alanine, aspartate, and glutamate metabolism; and purine metabolism were selected for qRT-PCR verification. The results showed that the qRT-PCR results were similar to the RNA-Seq results, although they were different in the difference multiples, the overall trend was basically the same, indicating that the transcriptome data were reliable (Figure 16).

## 4. Discussion

*S*. *hepiali* is a medicinal fungus isolated from fresh *Cordyceps sinensis*. It is also considered to be an excellent substitute for natural *Cordyceps sinensis* because of its similar biological activities, including anti-cancer, hypoglycemic, and immune regulation [13]. However, like other filamentous fungi, a series of irreversible strain degradations occur during the passage and preservation of the strain, which seriously affects the industrial production of the strain and its related products [27]. In this study, the strain showed degradation during subculture. Previous studies have shown that the virulence decline caused by strain degradation is a common phenomenon in many entomogenous fungi [28]. They penetrate the cuticle of the host by releasing and replicating enzymes such as chitinase, protease, and lipase in the mycelium, and then enter the host’s body while continuously consuming the host’s nutrients to meet their physiological needs and releasing toxins, eventually leading to the death of the host [29]. Obviously, virulent strains have a greater advantage in this regard. However, the decrease in virulence may lead to a decrease in the enzyme activity released by the strain and the ability to obtain nutrients, thus affecting the growth rate of the strain. In addition, the virulence and sporulation of the strain were proven to have a positive correlation in early studies [28]. Therefore, from the F8 generation onward, the signs of degradation in growth rate, fresh weight, dry weight, and sporulation of the strain may be closely related to the decrease in virulence of the strain during passage [29]. At the same time, the physiological processes such as the growth rate of the strain are regulated by multiple genes. Therefore, the gene mutation of the strain may also induce changes in the growth rate, fresh weight, dry weight, and sporulation of the strain during the passage [27,30].

The degradation of fungi is usually closely related to an increase in the reactive oxygen species (ROS) content in the body. ROS is mainly involved in cell signaling events and maintaining cell function in the body. However, more than a certain concentration will have adverse effects on the organism and produce serious cell oxidative damage [31]. GSH-Px, CAT, and SOD are a set of antioxidant enzymes with the function of scavenging ROS. In particular, SOD and CAT are considered to be the two most important types of antioxidant enzymes in the antioxidant system, which removes ROS produced by abiotic stress in the organism and averts oxidative damage, and they were significantly correlated with T-AOC [32,33,34]. In addition, MDA content is an important indicator for measuring the level of ROS and the degree of oxidative damage [31]. In this study, the content of antioxidant enzymes and T-AOC in different generations of strains gradually decreased from the F8 generation onward; oppositely, the content of MDA gradually increased from the F8 generation onward. This indicated that the antioxidant activity of the strain changed with the degradation of the strain, which was also observed in previous studies on *Cordyceps militaris* [35]. In addition, earlier studies have pointed out that the degradation of filamentous fungi is actually a process of strain senescence or aging which will eventually lead to the decrease or loss of the sporulation capacity and growth rate of fungi and the sharp increase of ROS concentration. This is, in fact, similar to the results of this study [9,36]. Therefore, the changes to the various antioxidant enzymes, MDA content, and T-AOC in the process of strain degradation may be related to the oxidative stress reaction caused by the imbalance of antioxidant system in the organism, which is actually a phenomenon of strain aging caused by cell damage [9,34].

Fungal metabolites can resist a variety of biotic and abiotic stresses and play an important role in the field of human health due to their wide range of biological characteristics [37]. Previous studies have shown that with the degradation of fungi, their metabolites will also change to varying degrees [38]. In this study, we identified a total of 1529 metabolites from three groups of samples, including 936 positive ion mode metabolites (ESI+) and 593 negative ion mode metabolites (ESI−), revealing the diversity of *S. hepiali* metabolites. Through PCA and OPLS-DA model analysis, the differences in metabolic profiles between normal strains and degraded strains were revealed, indicating that the synthesis patterns of metabolites between normal strains and degraded strains may be different. A total of 573, 598, and 173 DAMs were identified between F1 vs. F8, F1 vs. F11, and F8 vs. F11, respectively. Obviously, the number of DAMs in the group comparing degraded strains was significantly lower than that in other comparison groups. Early studies have shown that the degradation of many fungal strains occurs simultaneously with the decrease in the number of DAMs, which is consistent with the results of this study [12]. Further studies showed that the DAMs identified between the three different comparison groups were mainly attributed to carboxylic acids and derivatives, organooxygen compounds, benzene and substituted derivatives, fatty acyls, and prenol lipids. Carboxylic acids and derivatives are a class of bioactive components present in natural and non-natural compounds [39]. As the compounds with the highest content in the three comparison groups, many secondary metabolites in their categories have also been shown to have potential value in the medical field. For example, aspartate synthesis can inhibit the proliferation of tumor cells [40]. As a dietary essential amino acid, histidine is a precursor of carnosine in human muscles and brain and also plays an active role in skin protection [41]. In addition to being generally used as a chelating agent in the food and beverage industry, citric acid is also widely used in the field of biopharmaceuticals. For example, it can be used as a drug additive to treat postmenopausal osteoporosis in women [42]. The content levels of these active secondary metabolites in normal strains and degraded strains were high (Table S2), indicating that the strains still had high nutritional value and medicinal value after degradation. The identified DAMs were further analyzed by using the volcanic map drawn by univariate analysis. The results showed that there were 30 significantly up-regulated and down-regulated DAMs (8 up-regulated and 22 down-regulated) in the three different comparison groups which were worthy of attention and could be used as potential biomarkers to distinguish normal strains from degraded strains.

The KEGG (Kyoto Encyclopedia of Genes and Genomes) database is used to understand the advanced functions and uses of cells, organisms, and ecosystems from molecular level information. Therefore, KEGG pathway analysis is necessary to understand the function of metabolites and the relationship between metabolites. Earlier studies on *Cordyceps militaris* showed that the degradation produced by the strain during subculture was associated with a decrease in cordycepin content [12]. Subsequently, further studies showed that ABC transporters significantly promoted the synthesis of cordycepin [43]. ABC transporters can realize the transmembrane transport of polysaccharides, amino acids, and alkaloids in and out of cells, and their enrichment level in organisms may be related to metabolic activities [44]. Cordycepin is mainly derived from *Cordyceps* fungi and is a natural nucleoside analogue. It was first discovered in 1950 and is currently one of the main active ingredients in *S. hepiali*. However, the degradation of *Cordyceps* fungal strains accompanied by a decrease in cordycepin content has been demonstrated in many similar studies [12,13,45]. In this study, ABC transporters were significantly enriched in the comparison groups of F1 vs. F8 and F1 vs. F11 but not in the degenerated strain comparison group F8 vs. F11. This indicated that the decrease in cordycepin content may hinder the enrichment of antioxidant-related metabolites in ABC transporters during subculture, which in turn induced the degeneration of the strain. As a compound beneficial to human health, amino acids have the important effects of enhancing human immunity and promoting wound healing [37,46]. However, for filamentous fungi, the type of amino acid is also an extremely important factor affecting the growth rate of its mycelium [47]. For example, aspartate has been shown to significantly inhibit the growth of filamentous fungal hyphae in previous studies [47]. However, alanine, aspartate, and glutamate metabolism were significantly enriched in the F8 vs. F11 comparison group. Therefore, the enrichment of aspartate and its related metabolites during subculture may be one of the key factors inducing strain degradation.

The rapid rate of updating of transcriptomics technology has made it possible to speculate on the function of unannotated genes as well as to analyze in more depth the changes in gene expression in different organisms by means of this technology [48]. In this study, we performed transcriptome analysis on three different generations of strains and screened DEGs according to the significant levels of gene expression in different comparison groups. The results indicate that strain degradation affects the number of DEGs. In addition, the clustering pattern analysis of three different generations of strains showed that the F1 generation was clustered into one category, while the F8 and F11 generations were clustered into another category. Combined with the analysis results of physiology and metabolomics, the F8 generation should be the key stage for the degradation of the strain.

In recent years, high-throughput sequencing platforms combined with RNA-Seq technology have greatly promoted the research of molecular biology [49]. Therefore, this study used Illumina RNA-Seq technology to perform GO enrichment and KEGG enrichment analysis on DEGs of different comparison groups. GO enrichment analysis of DEGs showed that GO classification was mainly labeled as three functional categories: molecular function (MF), biological process (BP), and cellular component (CC). Further analysis showed that the catalytic activity, acting on DNA, was significantly enriched in both the F1 vs. F8 and F1 vs. F11 comparison groups, including many significantly up-regulated functional genes, *HRQ1* and *exo1*, for example. *HRQ1* helicase is a relatively conserved functional gene in the fungal genome, mainly involved in DNA inter-strand crosslink (ICL) repair, telomere maintenance, and maintenance of genomic stability [50,51]. *Exo1* is an evolutionarily conserved exonuclease, which is mainly involved in DNA metabolism in the genome. It plays an important role in replication stress response, double strand break repair, mismatch repair, nucleotide excision repair, and telomere maintenance [52]. However, in the comparison group of degenerated strains (F8 vs. F11), the significantly enriched GO terms were the MCM complex, structural constituent of ribosome, and spindle assembly. Minichromosome maintenance proteins (MCMs) were first discovered in *Saccharomyces cerevisiae*. Subsequent studies have found that such genes are widely present in eukaryotes and archaea [53,54]. However, earlier studies have shown that abnormal expression and activation of the MCM complex are usually accompanied by the occurrence of various malignant tumors and accelerate unstable and uncontrolled cell cycle progression in the genome [53]. The mitotic spindle is a special organelle composed of fiber structures and is also one of the most dynamic cytoskeleton structures. However, its maintenance requires continuous and uninterrupted energy consumption [55]. Obviously, the group (F8 vs. F11) comparing the degenerated strains was significantly enriched with GO terms and DEGs that were not conducive to the growth of the strains. In addition, the KEGG pathway enriched by DEGs was analyzed across different comparison groups. The results showed that DEGs in the comparison groups F1 vs. F8 and F1 vs. F11 were significantly enriched in three KEGG pathways: fatty acid degradation, mismatch repair, and DNA replication. Fatty acids are an essential component of the human body for establishing cell membranes. They are usually related to metabolism, can regulate enzyme activity, and are also a substrate for cytokine synthesis. Previous studies have found that they play an important regulatory role in different cell types [56,57]. The degradation process of fatty acids is mainly carried out through the ß-oxidation cycle, which produces acetyl-CoA and reduced NADH and FADH cofactors. At the same time, it is also inseparable from the genetic regulation system and the fatty acid transport system [58]. Aging is a degenerative state accompanied by tissue stem cell failure, cell senescence, and metabolic dysfunction. Earlier studies have suggested that aging is associated with genomic instability caused by DNA damage accumulation. Mismatch repair is one of the DNA repair mechanism networks evolved by organisms to cope with DNA damage. It can correct the mismatches that occur during DNA replication, remove the region containing the wrong base in the newly synthesized DNA strand, and resynthesize to ensure the integrity and stability of DNA [59]. As a carrier for storing genetic information, DNA needs to undergo an accurate replication in each cell cycle, thereby transmitting the genetic information of the parent to the offspring, and the cells finally divide to produce new cells when the conditions are met [60]. However, in the F8 vs. F11 comparison group, many DEGs that may adversely affect the normal physiological processes of the strain were significantly enriched and up-regulated in four KEGG pathways (pyrimidine metabolism, cell cycle yeast, DNA replication, and ribosome). For example, genes such as *mps1*, *mcm7*, and *mcm6* have been shown to be closely related to cancer in previous studies, and can increase the probability of canceration in normal cells. They are also significantly enriched and up-regulated in the F8 vs. F11 comparison group [61,62,63].

The correlation analysis of metabolome and transcriptome between different comparison groups showed that DAMs and DEGs were mainly enriched in ABC transporters; fatty acid degradation; alanine, aspartate, and glutamate metabolism; and purine metabolism. In the ABC transporters pathway, three significantly up-regulated genes (*Abcb11*, *Abcd3*, and *Abcb4*) were found to be associated with protein expression in the F8 and F11 strains. Early studies have found that such proteins are mainly involved in bile transport in the human body, but their susceptibility to genetic variation usually leads to the occurrence of cholestatic liver disease and gallstones [64,65]. Among them, high expression of the *Abcd3* gene has been shown to be associated with apoptosis and the occurrence of some cancers [66]. In addition, the content of deoxycytidine was higher in the F8 and F11 strains. Previous studies have shown that such metabolites can inhibit DNA methyltransferase and participate in the regulation of gene expression [67]. Therefore, the changes to DEGs in the ABC transporters pathway of the F8 and F11 strains may be related to the higher content of deoxycytidine in the degraded strains. At the same time, the significant upregulation of these DEGs in the degenerated strains may also affect the enrichment of compounds such as deoxycytidine and phosphoric acid in this pathway. The *Acsl1* gene is mainly involved in the synthesis of triglyceride (TG) and the oxidation of fatty acids in adipocytes. Overexpression of *Acsl1* usually results in a significant increase in triglyceride content in cells [68,69]. Recent studies have found that the *Acsl1* gene is a key enzyme in regulating lipid metabolism [70]. The *Acsl5* gene, which also belongs to the Acyl-CoA synthetase enzymes (ACSL) family, is a category of isozymes located in the mitochondria which is usually related to triglyceride synthesis and apoptosis [71,72]. Early studies have shown that some triglyceride compounds have antibacterial activity against Gram-negative bacteria, Gram-positive bacteria, and some specific fungi [73]. The *Acsl1* and *Acsl5* genes significantly enriched and up-regulated the fatty acid degradation pathway; in addition to their own characteristic of inducing apoptosis, the increase in the level of triglyceride content may also be related to strain degradation. Amino acids are the basic components that support life. They can promote protein synthesis and are beneficial to cell function [74]. However, recent studies have found that some amino acids may be associated with aging and age-related diseases [75]. In the alanine, aspartate, and glutamate metabolism pathway, many DEGs are primarily involved in the regulation of amino acid synthesis. For example, among the significantly up-regulated DEGs, the *Ass1* gene is a key enzyme in arginine biosynthesis, and the *Csad* gene may be regulated by sulfur amino acids [76,77]. Therefore, the two types of amino acids oxoglutaric acid and GABA, which were significantly increased in the degraded strains, may be related to gene regulation. Some of the enriched amino acids may be detrimental to the growth of the strain, such as aspartate [47]. Purine and its derivatives are mainly involved in intracellular energy homeostasis and nucleotide synthesis. As it is a kind of nutrient needed by fungi, many types of fungi can synthesize purine through specific methods [78,79]. The results of this study showed that the degenerated strains activated the purine metabolism pathway; significantly up-regulated the *Adk*, *Pgm1*, *Paics*, *Ak2*, *Ttr*, *Nme2*, *Aox3*, and *Uox* genes; and accumulated more purine metabolites, such as hypoxanthine, xanthine, and guanosine. The main function of *Ak2* is to catalyze the exchange of nucleotide phosphate groups and mediate mitochondrial apoptosis by forming the AK2-FADD-caspase-10 (AFAC10) complex [80,81]. In addition, the 13 significantly up-regulated or down-regulated DEGs identified in this study were proven to be involved in the regulation of many metabolites and metabolic pathways in earlier studies. For example, the *Pgm1* gene regulates glycogen and glycolysis energy metabolism by catalyzing the mutual conversion of glucose 1-phosphate and glucose 6-phosphate [82]. Therefore, 13 DEGs in the degenerated strains may be involved in purine metabolism and cause the accumulation of purine metabolites such as hypoxanthine, xanthine, and guanosine. Previous studies have found that many purine metabolites have adverse effects on the organism, including inducing apoptosis, causing DNA damage, and increasing ROS content [78,83]. In this study, the degradation of the strain led to the enhancement of the purine metabolism pathway, and many corresponding secondary metabolites were produced to inhibit the growth of the strain. Therefore, many genes and metabolites that induce apoptosis and the senescence of strains cause cell damage and consume a lot of energy, which ultimately leads to the inhibition of mycelial growth.

## 5. Conclusions

In this study, multi-omics analysis was used to explore the response of an *S. hepiali* strain to key metabolic pathways and its degradation mechanism during subculture. When the strain was cultured to the F8 generation, obvious signs of degradation began to appear, and the antioxidant enzyme system of the strain was destroyed, which also reduced the spore production of the strain and affected its growth and development. In addition, transcriptomics and metabolomics showed that strain degradation mainly affected ABC transporters; fatty acid degradation; alanine, aspartate, and glutamate metabolism; and purine metabolism pathways. In the process of subculture, many DEGs and DAMs that can induce apoptosis and senescence were significantly enriched, which was one of the key reasons for degradation.

## Figures and Tables

**Table 1:** Sequencing data and quality assessment.

Samples	Raw Reads	Clean Reads	Raw Bases (bp)	Clean Bases (bp)	Error Rate (%)	Q20 (%)	Q30 (%)	GC Content (%)
F11_1	59,297,640	58,657,916	8,953,943,640	8,842,076,476	0.001886	99.01	97.02	56.32
F11_2	61,246,102	60,561,152	9,248,161,402	9,122,703,173	0.001779	99.01	97.04	56.25
F11_5	52,317,986	51,644,146	7,900,015,886	7,783,025,546	0.001787	98.94	96.85	56.44
F1_2	70,055,490	69,094,666	10,578,378,990	10,412,699,146	0.007917	98.79	96.44	56.04
F1_4	50,425,330	49,984,240	7,614,224,830	7,537,756,194	0.002278	99.15	97.37	55.80
F1_6	52,412,658	51,744,950	7,914,311,358	7,796,427,041	0.002247	98.88	96.68	55.57
F8_1	65,251,706	64,482,778	9,853,007,606	9,715,403,210	0.001802	98.95	96.89	56.04
F8_4	67,137,558	66,349,958	10,137,771,258	10,001,417,170	0.001818	99.00	97.03	55.72
F8_6	55,659,734	55,055,572	8,404,619,834	8,297,541,185	0.001751	99.03	97.07	56.17

## Data Availability

The raw data were submitted to the National Center for Biotechnology Information Sequence Read Archive (NCBI SRA) (accession number: PRJNA1125860).

## Supplementary Materials

The following supporting information can be downloaded at: https://www.mdpi.com/article/10.3390/antiox13070780/s1, Table S1: The primers used for qRT-PCR; Table S2: Metabolites identified in different sample groups; Table S3: DAMs of F1 vs. F8; Table S4: DAMs of F1 vs. F11; Table S5: DAMs of F8 vs. F11; Table S6: Significantly enriched KEGG pathways in different generations of strains; Table S7: GO term analysis of F1 vs. F8 comparison group enrichment; Table S8: GO term analysis of F1 vs. F11 comparison group enrichment; Table S9: GO term analysis of F8 vs. F11 comparison group enrichment; Table S10: The common KEGG enrichment pathway of DAMs and DEGs in F1 vs. F8 comparison group; Table S11: The common KEGG enrichment pathway of DAMs and DEGs in F1 vs. F11 comparison group.
